# Fungi-Based Bioproducts: A Review in the Context of One Health

**DOI:** 10.3390/pathogens14050463

**Published:** 2025-05-09

**Authors:** Thais Kato de Sousa, Adriane Toledo da Silva, Filippe Elias de Freitas Soares

**Affiliations:** 1 Instituto de Patologia Tropical e Saúde Pública, Universidade Federal de Goiás, Rua 235 s/n, Goiânia 74690-900, GO, Brazil; thaiskato25@gmail.com; 2 Departamento de Química, Universidade Federal de Lavras, Lavras 37200-900, MG, Brazil; adrianetoledo123@gmail.com

**Keywords:** entomopathogenic fungi, biopesticides, biosafety

## Abstract

Entomopathogenic fungus-based biopesticides are an excellent alternative to synthetic pesticides and are widely used in insect pest control. With the transformations of the agri-food system, it is important to consider the One Health approach, which recognizes that health threats are shared at the interface between people, animals, plants, and the environment. The safety and environmental impact of fungi-based insecticides should be assessed comprehensively, taking into account not only their effects on non-target organisms and human health but also their environmental fate. This includes how these substances degrade, persist, or dissipate in soil, water, and air and their potential to bioaccumulate or leach into groundwater. Such assessments are essential to ensure that their long-term use does not pose unintended risks to ecosystems or public health. This systematic review aims to identify and analyze available studies on the potential One Health hazards associated with fungal biopesticides. A total of 134 articles were selected: 84 bioassay articles (63%), 36 case reports (27%), 10 field studies (7%), and 4 other types of studies (3%). Of these articles, 59 were studies on vertebrate animals and 65 studies on invertebrate animals, 6 studies on diverse organisms, 2 studies focused specifically on risk assessment for non-target organisms in the environment, while 2 other studies looked at the toxicological hazards associated with human exposure to the metabolites of the fungus present in air. The United States had the highest number of publications (33). *Beauveria bassiana* and *Metarhizium anisopliae* followed by the fungi *Cordyceps fumosorosea* (*Paecilomyces fumosoroseus*) and *B. brongniartii* were the most prevalent fungal species in the studies. This review highlights that case reports of infections in humans and other vertebrates by fungi are not related to the use of fungal biopesticides. A predominance of studies with bees was identified due to the importance of these insects as pollinators. The findings indicate that fungal biopesticides pose minimal risks when used appropriately. Nevertheless, the necessity for standardized safety assessments is emphasized. In order to ensure greater effectiveness, it is essential to develop unified protocols and bioassays with specific risk indicators aligned with the One Health approach. This includes evaluating potential effects on pollinators, vertebrate toxicity, and the environmental persistence of metabolites. In future research, the development of integrated guidelines that simultaneously consider human, animal, and environmental health is recommended.

## 1. Introduction

Population growth, urbanization, and rising incomes have increased the demand for food. Population projections indicate accelerated and continuous growth in the coming decades [1]. The use of pesticides has been intensified to combat arthropods, microbial pathogens, and weeds, with the justification of increasing food production to feed a constantly growing population [2]. However, this linear relationship between population growth and food demand is accompanied by the challenge of producing sustainably [3].

The growing concern about environmental issues related to the use of chemical pesticides has led to the search for new technologies to act as substitutes in pest management [4]. In view of this problem, biological control has arisen as a viable alternative and a sustainable and effective method [5].

Biological control is a naturally occurring ecological process in which natural enemies regulate pest populations without human intervention. However, humans can enhance or manipulate this process through the intentional introduction, conservation, or augmentation of biological control agents to improve pest suppression outcomes [6].

Nowadays, when integrated pest management (IPM) is increasingly being used towards sustainable agriculture, biological control is becoming increasingly important [7]. Biological control can be defined in the context of reducing populations of undesirable organisms that occur in agroecosystems through the use of natural enemies such as predators, parasitoids, parasites, herbivores, competitors, and pathogens to control agricultural pests [6]. Biocontrol with entomopathogens uses microorganisms, such as fungi, bacteria, viruses, protozoa, and nematodes, to control insect pests [8].

Entomopathogenic fungi have a broad spectrum of action and are capable of colonizing various insect species [9]. The use of these fungi in agriculture is growing, mainly due to the expansion of organic agriculture and the increasing demand for food free from pesticide residues. These biological control agents are important tools in integrated pest management programs in the current agricultural scenario [10]. The fungi *M. anisopliae* and *B. bassiana* are among the most used in insect pest biological control programs [11].

**Figure 1:**
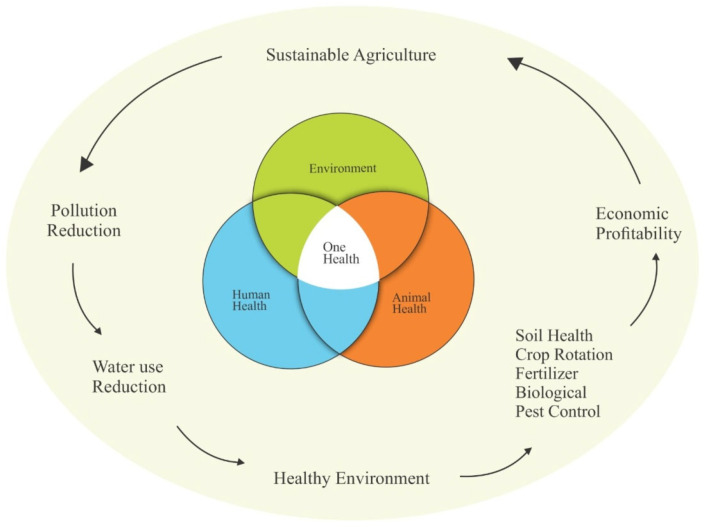
One Health and sustainable agriculture approach.

Biological pest control products are regulated in several countries and are evaluated through studies and tests required by laws and regulations [12,13]. In this context of transforming the agri-food system with the use of sustainable agricultural practices, it is important to consider the One Health approach, which recognizes that health threats are shared at the interface between people, animals, plants, and the environment [14]. The term can also be defined as a global concept of promoting human health based on a strategy for better understanding current health problems created by interactions between humans, animals, plants, and the environment [15]. This interdependence between living beings, especially in relation to land and water, is intrinsically linked to sustainable agriculture (Figure 1).

Therefore, the evaluation of fungal bioproducts from a One Health perspective covers two crucial and interrelated aspects: (1) the assessment of effects on non-target organisms, including pollinators, soil microorganisms, and aquatic life, with a view to ensuring ecological safety; and (2) the analysis of potential health risks, such as exposure to toxic fungal metabolites or airborne spores, which can affect humans and animals.

In general, concerns about the use of biological control agents are related to the possible impacts of using these products on the environment and on the exposed population [13]. In the case of a biological agent, there could be consequences of using these products, such as toxin production, irritability, allergenicity, and the potential for pathogenicity [13,16]. In order to better understand whether these interactions may exist with the use of fungal bioproducts, this review aims to describe the main genera of fungi used as biopesticides; identify and discuss the available studies on the potential of fungal biopesticides to cause any danger to One Health; and, finally, through this analysis, identify the main knowledge gaps in relation to the review topic.

## 2. Methods

This study was conducted using the Reporting Items for Systematic reviews and Meta-Analyses (PRISMA) for Scoping Reviews (PRISMA ScR) guidelines, a guide developed to standardize the reporting of reviews [17,18]. Scoping reviews, a type of knowledge synthesis, follow a systematic approach to map the evidence on a topic and identify key concepts, theories, sources, and knowledge gaps [18]. To develop this review, a PRISMA ScR checklist was used, which contains 20 items that help authors develop and report a scoping review [18] (Table S1).

**Figure 2:**
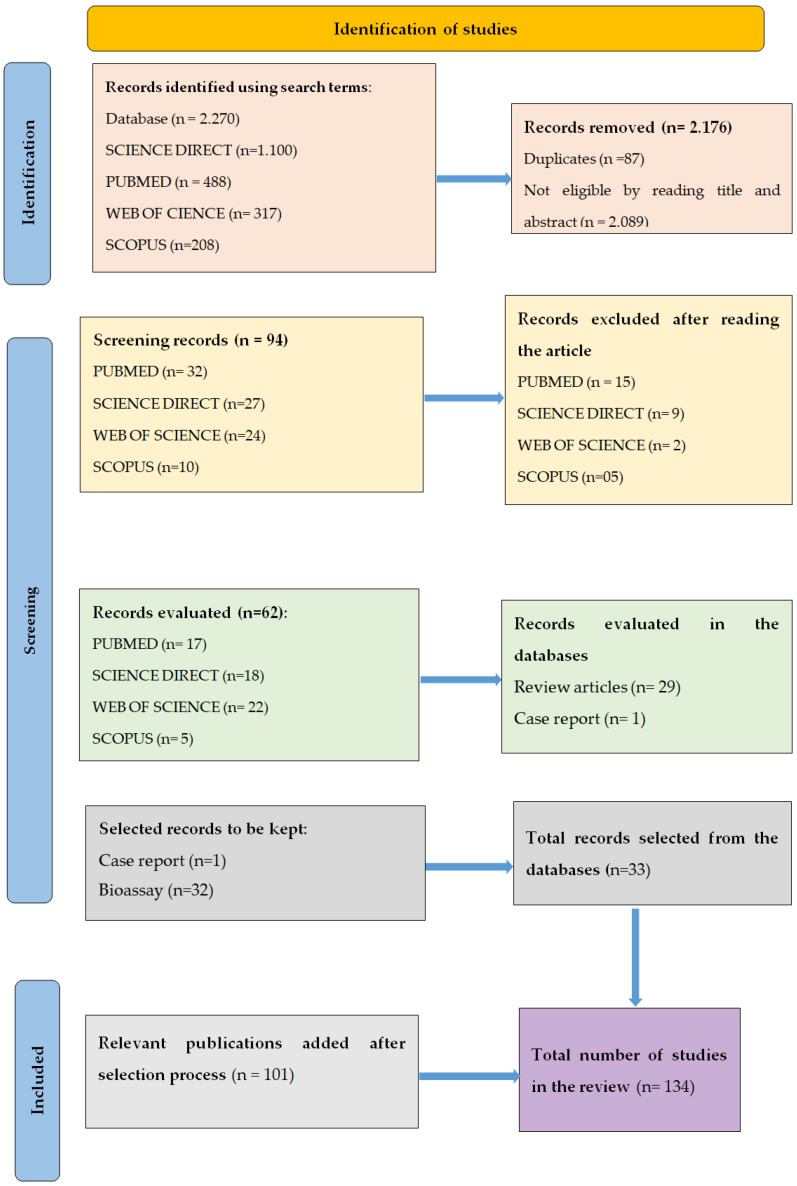
Registration flowchart for systematic reviews in databases (PRISMA 2020).

Initially, 2270 articles were identified by means of search terms in the following electronic databases: Science Direct presented the largest number of articles (1100), followed by the PubMed database (488) (Figure 2). Eighteen search term combinations were used using the following keywords: “biological control”, “biocontrol”, “biosafety”, “entomopathogenic fungi”, “fungi”, “microbiology”, “nematophagous fungi”, “infection”, and “safety” (Table 1). The terms were selected due to their importance to the thematic areas highlighted in this study: biopesticides, risk assessment, and One Health.

The selection of articles in each database was carried out by means of titles and abstracts, to obtain the full texts. When the title and abstract were not considered clear, the article was searched in its entirety to avoid the risk of leaving important studies out of this review [19]. However, at the end of this process, it was identified that almost half of the studies identified in the databases were review articles, and it was decided to search for the primary articles cited by these review studies. The relevant articles for the research were added manually.

The articles identified in the initial search strategy were evaluated by two researchers. In this evaluation process, each researcher recorded whether or not they agreed with the inclusion of the article, based on the evaluation of the title and abstract. All articles screened in the previous phase had their eligibility confirmed by reading the full text of the article. Eligibility confirmation was performed by the pair of reviewers, and discordant opinions were resolved by presenting a plausible justification for keeping or excluding each study [20].

The Web of Science, Scopus, and Scielo databases accounted for 317, 208, and 157 articles, respectively. In total, 2176 articles were excluded: 87 duplicate articles in the databases, and 2089 were removed by reading the title and abstract (Figure 2). The study inclusion criteria were as follows: articles in English and with complete research results, available in the databases until 31 December 2022. The exclusion criteria were as follows: theses, dissertations, and abstracts of scientific events, technical reports, duplicate publications, and articles not written in English, articles that do not address the topic, and review articles.

A total of 94 studies were analyzed after reading and evaluating the articles in full, and 61 were excluded for not meeting the inclusion criteria. Thirty-three articles were selected in the databases. One hundred and one (101) relevant studies cited in this review were added manually. In total, 134 articles were selected for the systematic review (Figure 2).

To organize this review, a bibliometric analysis of the articles selected to compose the review was carried out. Bibliometrics consists of analyzing scientific or technical activity through quantitative studies of publications. Quantitative data are calculated from statistical counts of publications. It is a flexible method for evaluating the typology, quantity, and quality of information cited in research [21].

The process of extracting data from the eligible articles was carried out by collecting the following information and characteristics of the studies: 1. Fungus; 2. Study targets; 3. Effects; 4. Study type; 5. Reference/Year; and 6. Observations, with the most relevant aspects of this study. The results are presented in the form of tables, graphs, maps, and figures prepared using the following programs: Microsoft Excel 2016, CorelDRAW 2020, MapChart, Google Sheets online, and Free Word Cloud Generator. To organize the references, a bibliographic reference manager from the Federal University of São Carlos was used, available for free (MORE: Online Mechanism for References, version 2.0. Florianópolis: UFSC. Available at: http://www.more.ufsc.br/, accessed on 20 March 2023).

## 3. Results

### 3.1. Types of Study, Location, Fungal Species, Year of Publication, and Most Frequent Journals

**Figure 3:**
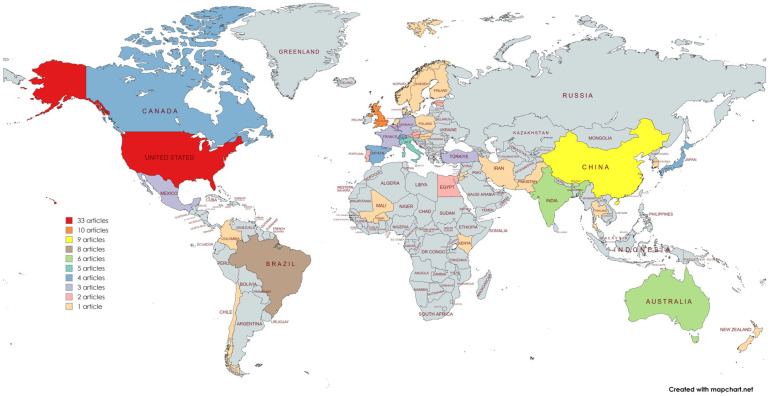
Geographical distribution of the origin of the studies included in this systematic review.

The studies were mainly conducted in North America, with 33 studies identified in the United States. In Europe, the United Kingdom stood out with 10 studies; in Asia, China accounted for 9 studies; and, in South America, Brazil appeared with 8 scientific productions. Countries like Australia (6), India (6), and Italy (5) also produced a significant number of studies (Figure 3).

**Figure 4:**
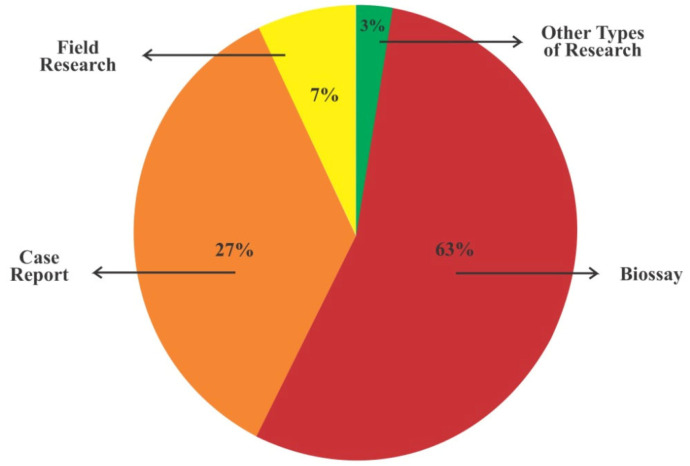
Types of methodological studies identified in this systematic review.

As for the selected study types, 84 bioassay articles (63%), 36 case reports (27%), 10 field studies (7%), and 4 other types of studies (3%) were identified (Figure 4).

**Figure 5:**
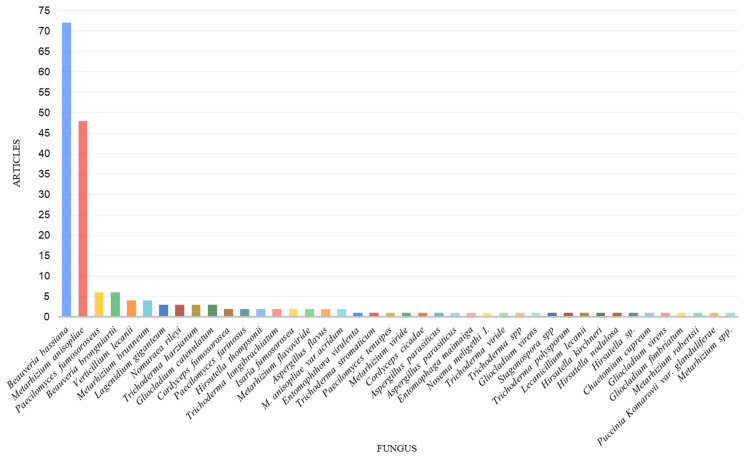
Relation between the number of articles in this review and the species of entomopathogenic fungi identified in each study.

Publications with the largest number of studies on the fungi *B. bassiana* (72) and *M. anisopliae* (48) prevail, followed by *C. fumosorosea* (*P. fumosoroseus*) (6) and *B. brongniartii* (6) (Figure 5).

**Figure 6:**
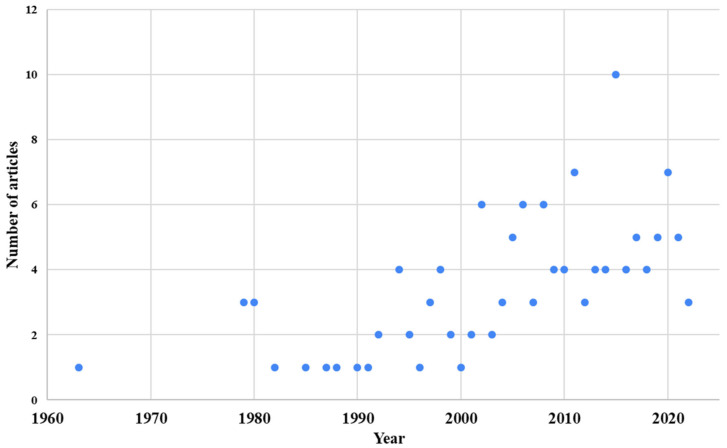
Number of articles selected on entomopathogenic fungi and their ability to cause any danger to One Health, by year of publication.

The frequency of studies found was higher from 2000 to 2020. The year 2015 had the highest number of publications (10) selected, with the oldest study from 1963 (1) and the most recent from 2022 (3) (Figure 6).

**Figure 7:**
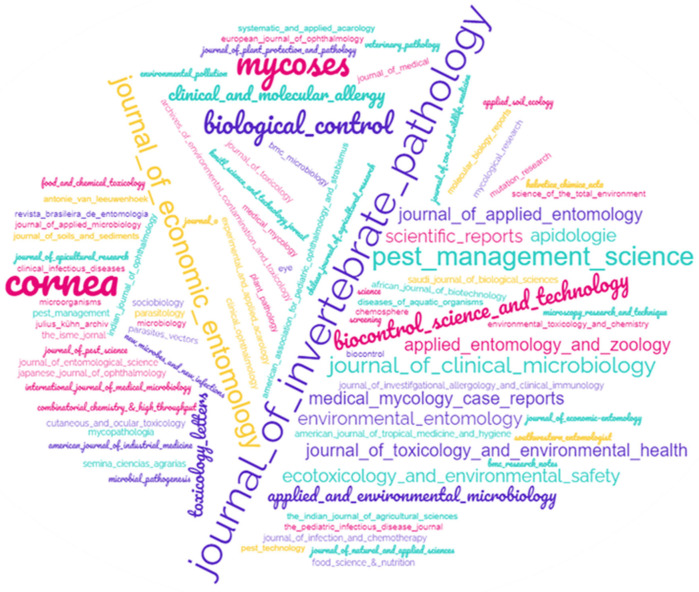
Word Cloud of journals that have published the most articles on fungi entomopathogenic fungi and their ability to have an effect on Public Health.

To identify which scientific journals published the most articles, a word cloud was generated (Figure 7). The most frequent periodicals were the *Journal of Invertebrate Pathology* (9) followed by the *Journal of Economic Entomology* (6), *Biocontrol Science and Technology* (5), and the *Journal of Clinical Microbiology* (5). Other journals such as *Cornea* (5), *Environmental Entomology* (4), *Mycoses* (4), *Medical Mycology Case Reports* (4), *Biological Control* (3), and *Pest Management Science* (3) also had notable frequencies on the map (Figure 7).

### 3.2. Studies on Vertebrate Animals

In total, 59 articles were identified related to the effects of entomopathogenic fungi used as biological control agents in vertebrates. Thirty case studies report infections in humans, with most cases related to eye diseases such as fungal keratitis (16), fungal sclerokeratitis (3), bullous keratopathy (1), and necrotizing scleritis (1) (Table 2). Four (4) studies are related to effects in humans: serum human allergenicity test and human lymphocyte toxicity [22,23,24,25].

Twenty-five articles related to the effects of fungal entomopathogens on other vertebrate animals are listed (Table 2). Six case reports were related to infections in animals such as cats, turtles (*Testudo elephantopus*, *T. gigantea elephantina*, *Terrapene carolina*, and *Trachemys scripta*), alligators (*Alligator mississipiensis*), chameleons (*Chamaeleo calyptratus* and *Furcifer pardalis*), three of which were captive animals, and one was a domestic animal [56,57,58,59,60]. From the articles on bioassays, 19 studies were carried out on animals such as mice (*Mus musculus*), rats, BALB/c mice, birds, pheasants (*Phasianus colchicus*), quails (*Coturnix coturnix*), rabbits, fish (*Melanotaenia duboulayi*, *Ulmerophlebia* spp., and *Ceriodaphnia dubia*), fish embryos (*Menidia beryllina*), and lizards (*Pogona vitticeps* and *Acanthodactylus dumerili*) (Table 3).

### 3.3. Studies on Invertebrate Animals

We identified 65 articles related to the effects of entomopathogenic fungi used as biological control agents on invertebrate animals. Of these articles, 55 are bioassay studies, and 10 are field studies. Thirty-five articles report studies carried out with bees, and the main species studied is *Apis mellifera* (24) (Table 4). In addition to bees, various insect species such as beetles, beneficial insects (ladybugs, some species of collembolan, aphids, and mites), crustaceans (shrimps), and earthworms have been analyzed in studies on the effects of fungal biopesticides on non-target organisms [81,82,83,84,85].

### 3.4. Experimental Studies on Various Organisms

Six selected articles are experimental studies carried out on different organisms such as bacteria, protozoa, plants, algae, microcrustaceans, crustaceans, insects, fish, frogs, rats, and human cells. Toxic effects were only observed in some plant species, insects, crustaceans, shrimp embryos, frogs, and fish (Table 5).

### 3.5. Other Types of Studies

Two selected articles refer to studies evaluating airborne exposure to fungal entomopathogens: the viability of airborne conidia in closed environments and analysis of bioaerosols applied to tomato greenhouses after using a fungal biopesticide [151,152]. Another two articles refer to toxicity tests of fungal secondary metabolites: oosporein, gliotoxin, and destruxins B, D, and E (Table 6).

## 4. Discussion

Interest in the use of entomopathogenic fungi in agriculture has increased significantly in recent years, given the problems inherent in the use of pesticides. Many integrated management strategies and research projects use entomopathogenic fungi due to the action characteristics of these pathogens, as they act by contact and ingestion and are found in nature, and it is more difficult for insects to become resistant due to their great genetic variability [155,156]. 

In this sense, it is important to note that biopesticides have specific registrations and regulations that vary between different countries [157]. There is growing concern worldwide about the use of biological agents in pest control and the possibility that these organisms could negatively interfere with beneficial organisms that coexist with the target pest in the same or neighboring ecosystems, and their possible adverse impacts on biodiversity and human health itself [158]. In this review, it was possible to identify that the oldest studies were case reports of entomopathogenic fungi infection in vertebrate animals, followed by reports of infections in humans and more recent studies evaluating the effects of fungal biopesticides on vertebrate and invertebrate animals [26,56,80,86,143,144].

The most important and common entomopathogenic fungi are found in the order Hypocreales, especially the species *B. bassiana* and *M. anisopliae*, which are the main fungi used in commercial formulations for biological control [156,159,160]. *B. bassiana*, *M. anisopliae*, followed by the fungi *P. fumosoroseus* and *B. brongniartii*, were the most prevalent fungal species in the studies (Figure 5). 

*Beauveria* spp. are filamentous fungi, and the most important species of this genus are *B. bassiana*, *B. brongniartii*, *B. morpha*, and *B. caledonica* [161]. *M. anisopliae* is a fungus belonging to the phylum Ascomycota and is the most studied species of the genus *Metarhizium* [162]. The fungus *P. fumosoroseus*, currently known as *C. fumosorosea*, is an active ingredient in some commercial products worldwide [156,163,164] The taxonomic classification of this genus has undergone numerous changes over the last few years. The species pathogenic to arthropods were grouped into the genus *Isaria*, and, then, based on multigenic analysis, most of them were transferred to the genus *Cordyceps* [156].

Overall, the use of biopesticides has increased significantly worldwide. The market for biological products for agricultural use is mainly concentrated in higher-income countries located in North America and Western Europe, regions where the biopesticide market is well established and on an upward trend, mainly due to the need to implement sustainable agricultural practices [165]. In this regard, it is interesting to note that the origin of the scientific productions selected for this systematic review was also concentrated in the United States and the United Kingdom.

In Latin America, Brazil also featured prominently in the scientific productions selected, with eight articles making up this systematic review [166]. The Brazilian market also has a number of biological products; currently, 146 products based on *Bacillus* spp., *Clonostachys rosea*, and *Trichoderma* spp. are registered as biofungicides; 3 products based on *B. subtilis* are registered as biobactericides; 155 products based on *B. amyloliquefacies*, *B. licheniformis*, *B. methylotrophicus*, *B. paralicheniformis*, *B. subtilis*, *B. velezensis*, *P. lilacinus*, *Pasteuria nishizawae*, *Purpureocillium lilacinum*, *T. endophyticum*, and *T. harzianum*, among others, are registered as bionematicides [167]. The fungi *B. bassiana* and *M. anisopliae* are also widely used to control agricultural pests [165]. As bioinsecticides, 95 products based on *B. bassiana* and 91 products based on *M. anisopliae* have been registered [166].

## 5. Effects on One Health

### 5.1. Human Health

Of the 134 articles analyzed, 30 case studies reported fungal infections in humans, and 4 studies were related to the allergenic potential of fungal biopesticides (Table 1). There was a predominance of reports of infections caused by the fungi *B. bassiana* (14), *M. anisopliae* (12), *T. longibrachiatum* (2), *A. lecani* (1), and there was one article in which it was not possible to differentiate between the species *B. bassiana* or *B. brongniartii* (Table 2).

Nine articles were case reports of fungal keratitis caused by *B. bassiana* in women (Table 1). Three of these reports of fungal keratitis caused by *B. bassiana* were of women who wore contact lenses, one of whom was an occasional farm worker who used inhaled corticosteroids and the other who tended a rose garden [37,42,51]. Five reports of *B. bassiana* fungal keratitis were of women with previous diseases such as diabetes, hypertension, corneal dystrophy, a history of antibiotic and corticosteroid use, and one report of previous corneal transplant surgery [31,39,42,46,49]. In addition, a case of necrotizing scleritis and endophthalmitis was reported with a history of ocular trauma in a rice plantation, an epithelial defect in the sclera, and the use of systemic immunosuppressive therapy [41].

Three case reports were of men with an atypical presentation of fungal keratitis caused by *B. bassiana*: the first case presented infection after the removal of a foreign body and treatment with antibiotics and corticoids; the second case, a rural worker; and the third, a case of infection after accidental contact with rainwater [26,47,55]. 

Six studies were reports of fungal keratitis caused by *M. anisopliae* (Table 2). Four case reports of fungal keratitis caused by *M. anisopliae* had a history of contact lens use, three of which were women, and one was a 12-year-old child [32,43,52,54]. Two reports were of healthy young adult men, one of whom had a history of a dirt-blasting accident in the eye while gardening [27,48]. Three cases were of scleroceratitis caused by *M. anisopliae*, two women and one man, one living in a rural area, another woman with a report of an accident to the eye while mowing the lawn, and an elderly rural resident with a history of rheumatoid arthritis and systemic corticosteroid use [38,50,53].

*M. anisopliae* and *B. bassiana* are two cosmopolitan species, insect pathogens that grow naturally in soils and are found in almost every ecosystem in the world [161,168]. Despite this, cases of fungal keratitis caused by *M. anisopliae* and *B. bassiana* are not common [51,52]. Most of the keratitis cases selected in this review are not from immunocompetent people, and the eye infections observed were not related to the use of commercial fungus-based products for biological pest control. 

Fungal keratitis, also known as keratomycosis, is caused by the presence of fungi that can lead to serious eye infections, especially when the cause is related to eye trauma, drug abuse, or low immunity [169,170]. It is an infection that mainly affects agricultural workers or individuals involved in outdoor activities [51,52]. Risk factors include allergic conjunctivitis, previous eye surgery, and previous treatment with broad-spectrum antimicrobial agents, corticosteroids, and contact lens use [51].

Although it is a rare event, under immunosuppression, systemic human infection by *B. bassiana* is possible [33]. Three cases of fungal infection have been reported in cancer patients: one case of empyema by *B. bassiana* in a man with lung adenocarcinoma and two cases of systemic fungal infection by *B. bassiana* in patients with acute lymphoblastic leukemia [33,34,36]. A case of systemic fungal infection by *M. anisopliae* was also reported in a 9-year-old child with acute myeloid leukemia, who died [29]. Another pediatric report identified was a case of cutaneous infection by the fungus *M. anisopliae* in thigh ulcers in a child with ectodermal dysplasia [40]. 

Opportunistic fungal pathogens only cause infection when there are breaches in the protective barriers of the skin and mucous membranes or when failures in the host immune system allow penetration, colonization, and production in the host [171]. Invasive disseminated systemic infections can occur when the innate cellular immune response is impaired under conditions such as steroid treatment, prolonged granulocytopenia following chemotherapy, hematological malignancies, or bone marrow transplantation [33]. In this context, invasive fungal infection (IFI) is an important cause of hospitalization and mortality among leukemia patients. In 92 hospitalized patients with leukemia, the prevalence of invasive fungal infection was 27.17% (25 cases), and the main fungal species found were *Candida* and *Aspergillus* spp. [172]. 

Still on the studies selected in this review, three cases of respiratory infections were reported: one report of sinusitis by *M. anisopliae* in immunocompetent patients (a man and a woman); one report of sinusitis; and another of rhinosinusitis by *T. longibrachiatum*, the first infection in a transplanted patient using corticoids and the second in a young immunocompetent patient [28,30,45]. Fungi of the genus *Trichoderma* are also used to control various plant diseases of agricultural importance, and the species *T. longibrachiatum* has great potential for use in biological pest control [173]. Generally, healthy and immunocompetent individuals have high innate resistance to fungal infection, despite being constantly exposed to infectious forms of various fungi present as part of the endogenous microbiota or in the environment [171].

It is important to note that, in most of the case reports in this review, the fungi were identified through culture. Only two studies in this review performed fungal identification through PCR (polymerase chain reaction)-based DNA sequencing analysis: a case report of fungal infection by B. bassiana and a bioaerosol analysis study that identified the fungal strain of a biocontrol product based on *T. harzianum* [55,152].

In recent years, innovations in fungal identification have reduced the exclusive reliance on morphological characteristics, which were previously used to distinguish species. Currently, morphology still assists in genus-level identification, but species differentiation is primarily achieved through DNA sequencing, by comparing specific genomic regions with reference databases [156]. 

*Akanthomyces lecani* is a pathogen used to control arthropods and plays an important role in regulating populations of phytophagous insects [174]. An epidemiological cohort study of 329 Danish greenhouse workers assessed specific sensitization to extracts of commercial preparations of *V. lecanni* and showed that exposure to this biopesticide can confer a risk of specific sensitization mediated by immunoglobulin E (IgE) [35]. 

Some studies have also evaluated the allergenic potential of *B. bassiana* and *M. anisopliae*, and it has been possible to identify a greater hypersensitivity to these species in patients who already have allergies to fungi [22,23,24]. Human exposure levels and sensitization thresholds are unknown for most allergens, including fungal biopesticides. These factors should be considered critical when assessing the risk of developing human allergic diseases [73]. The handling of the fungus during manufacture, storage, and field application can expose workers to very high doses of fungus [71]. In rural areas, the use of fungi in agricultural pest control can greatly increase the potential for human exposure to these agents [23].

The toxic effects of secondary metabolites of entomopathogenic fungi, such as beauvericin (*B. bassiana*), oosporein (*B. brongniartii*), gliotoxin (*A. verrucaria* (*G. fimbriatum*)), and destruxins B, D, and E (*M. anisopliae*), were analyzed in three studies (Table 2 and Table 6). The results indicated that beauvericin is genotoxic to human lymphocytes in vitro, causing a significant increase, depending on the concentration, in chromosomal aberrations, sister chromatid exchanges, and micronuclei. This metabolite also significantly decreased the mitotic index at the two highest concentrations [25]. From a physicochemical analysis study, the metabolite oosporein can hardly be absorbed into organisms and pass through the biological membrane [153]. In mutagenicity assays, no genotoxicity was observed for the secondary metabolites: oosporein, gliotoxin, and destruxins B, D, and E [154].

The use of species from the genus *Gliocladium* as biocontrol agents has been widely studied and applied in agriculture, particularly for the control of phytopathogens and diseases in various crops [175,176,177]. In addition, these species have shown potential in suppressing post-harvest diseases, such as anthracnose in tomatoes [178]. These applications highlight the importance of studying the toxicity of their metabolites, given their increasing use in sustainable agriculture.

### 5.2. Animal Health

#### 5.2.1. Vertebrate Animals

The oldest case reports selected in this review are of pulmonary infections in reptiles such as turtles, crocodiles, and alligators, and the main etiological agents are *B. bassiana* (three articles) and *M. anisopliae* (one article) (Table 3). Three of these reports were from captive animals, and the low temperatures may have favored fungal [56,57,58]. There have been reports of mycosis (glossitis, stomatitis, pharyngitis, and visceral mycosis) by *M. anisopliae* in chameleons and lizards kept as pets, fungal pneumonia by *M. anisopliae* in a free-living American alligator with anorexia and abnormal buoyancy, and a report of invasive rhinitis by *M. anisopliae* in a domestic cat [59,77].

Entomopathogenic fungi require environmental conditions of high relative humidity, moderate temperatures, and protection from solar radiation in order to germinate and consequently cause disease in susceptible hosts [157]. An important factor for the survival of entomopathogenic fungi in the field is temperature. High temperatures impair the survival of the fungus, while low temperatures increase its persistence [169].

Some bioassay studies to assess toxicity in non-target organisms (mice, rats, mice, rabbits, lizards, birds, rats, and fish) used fungal preparations at doses higher than those applied in field practice (Table 3). The effects on non-target organisms at high fungal concentrations of *B. bassiana, M. anisopliae, P. fumosoroseus* (= *C. fumosorosea*), and *H. thompsonii* ranged from no effects to death, weight loss, rupture of embryos, and transient liver changes [61,62,64,67,70,71].

The study conducted by Ward et al. (2011) [73] used a fungal concentration estimated within a possible human exposure range and found that *M. anisopliae* in mice induced a significant increase in IgE, suggesting an induction of an allergic response that may play a role in the development of asthma. However, the author points out that human exposure levels and sensitization thresholds are unknown for most allergens, including biopesticides. The study conducted by Alves-Filho et al. (2011) [74] used the fungus *T. stromaticum*, a biocontrol agent for the cocoa witches’ broom pathogen used in Brazil. The spores of *T. stromaticum* exerted an immunosuppressive effect in a murine model, preventing the expression and production of inflammatory mediators in macrophages (pro-inflammatory regulatory cytokines IL-10 and IFN-γ).

Biosafety assays must be evaluated to avoid possible risks to agricultural workers and the public during the production and application process, as well as to preserve the integrity of ecosystems [79]. Fish are a sensitive indicator for studying environmental, toxicological, and safety aspects of entomopathogenic fungi [80]. Older studies that still considered *Lagenidium giganteum* as a fungus (Table 4) showed that this parasite can affect some non-target aquatic species, including fish [145,146]. *L. giganteum* is a member of the class Oomycetes and is not a true fungus but is effective against larvae of the most harmful mosquito species [179]. 

The most recent studies are evaluations of toxicity and safety in non-target organisms. Sayed Ali et al. (2022) [79] investigated the biosafety of two bioinsecticides based on *B. bassiana* and identified changes in the function and size of the liver of rats that received a single oral dose of the products tested. The study conducted by Abrar et al. (2022) [80] evaluated the efficacy of the entomopathogenic fungus *A. parasiticus* against *Aedes aegypti* and the toxicity of the fungus on a non-target aquatic organism (*Hypophthalmichthys molitrix* fry). Cracked eyes, bleeding in the fins, and infection of the scales were the main morphological effects observed in the fry during the study. The results revealed that, although *A. parasiticus* is highly pathogenic to the dengue vector, it also has significant effects on organisms other than insects, and its application as a biological control agent requires safety considerations [80].

This review also included two articles on entomopathogenic fungi used in traditional Chinese medicine: *C. tenuipes* (*P. tenuipes*) and *I*. cicadae (C. *cicadae*) (Table 2). The toxicity potential of *C. tenuipes* was assessed in mice and rats, and no abnormalities were observed. *P. tenuipes*, also known as *I. tenuipes*, is a pathogenic fungus that infects pupae or larvae of various insects [75]. The safety assessment of *C. cicadae* in mouse tests showed that the oral administration of mycelia is also safe [78].

A study on the dermal toxicity of the entomopathogenic fungus *C. fumosorosea* (*I. fumosorosea*) in rabbits was carried out in Mexico, and there were no clinical signs of the disease in the treated animals following direct application of the fungus to the animals’ skin. The dermal toxicity test is one of the tests required by Mexican regulations in order to provide information on health risks, taking into account the periods in which workers are in direct contact with the microbial agent when applied [76,163]. 

#### 5.2.2. Invertebrate Animals

With regard to studies on invertebrate animals, 35 (53%) of the articles selected were studies on bees, especially the *A. mellifera* species. In this context, the importance of bees for the economy goes far beyond the production of honey. Pollination by different species, but, above all, by bees, is an essential process for plant production, whether in agricultural crops or forests, directly affecting food security [180]. 

The occurrence of fungal diseases in useful insects such as bees, silkworms, and natural enemies of pests can directly affect the survival of these organisms. The negative effects of the interactions between entomopathogenic fungi and natural enemies should be avoided, while the positive effects of the dispersal of entomopathogens should be valued, since natural enemies can increase the dispersal of fungi, especially under greenhouse conditions [157]. 

In the context of entomovement, bees that distribute biopesticides are exposed to high risks due to the side effects of these products. One study analyzed the potential adverse effects of a bioinsecticide based on the fungus *B. bassiana* on bees of the *Bombus terrestris* species and found that the bioinsecticide caused a decrease in bee survival [123]. Entomopathogenic preparations of a biofungicide containing *C. rosea f. catenulata* (*G. catenulatum*) spores and two bioinsecticides containing *B. bassiana* and *M. brunneum* affected the longevity of *A. mellifera* and *B. terrestris* bees. This evidence should be taken into account when developing new microbial preparations for systems that use pollinators as entomovectors for pest control [131]. A trial was conducted to determine the optimal concentration of a commercial formulation of *B. bassiana* vectored by the pollinator *B. impatiens* for the control of whitefly on greenhouse tomatoes. The medium concentration was selected as the optimal concentration because it provided the best pest control with the least impact on bees [106].

In addition to the risks to pollinators and entomovectors, entomopathogenic fungi can also affect predators and parasitoids, particularly when they come into contact with prey or hosts previously treated with these microorganisms [181]. Reports in the literature indicate that species such as *Harmonia axyridis* avoid feeding on prey infected with these fungi, which may reduce their effectiveness in biological control [182]. Furthermore, the introduction of exogenous fungal species has the potential to alter the soil microbial balance, for example, by stimulating or suppressing nitrogen-fixing bacteria, actinobacteria, and cellulose-degrading microorganisms [183,184].

It was possible to identify the relationship between the toxic effects on invertebrate animals and the different concentrations of the entomopathogenic fungi used in the bioassay studies, using formulations with fungal concentrations normally used in the field, dosages higher than those used in the field or recommended by the manufacturer, and concentrations lower than those used in the field [117,121,128,133].

The commercial entomopathogens *M. anisopliae* and *B. bassiana* reduced the survival of *A. mellifera* worker bees in four bioassays tested (spraying, contact on smooth surfaces, contact on soybean leaves, and mixing with jam paste), and the concentrations applied were higher than the dose recommended for use in the field [133]. In another study, *B. bassiana* and *M. anisopliae* caused a reduction in the survival of *A. mellifera* worker bees using various application methods at the concentration recommended by the manufacturer [138]. Bioassays were conducted to examine the pathogenicity of *B. bassiana* (four spore concentrations) against a predatory insect and its impact on beneficial arthropods, including *A. mellifera*. Higher mortality of beneficial arthropods was observed when exposed to the highest concentrations of *B. bassiana* [129]. 

The different results found in the bioassays with bees may have been influenced by the different concentrations of the fungi applied in the studies [133]. In this sense, standardized bioassays with the target pest are, in practice, the parameters evaluated in the laboratory that are most correlated with the success of the biopesticide when used by the end user. In order to obtain reliable data, various doses can be evaluated [156]. The concentrations of these preparations vary between different manufacturers, but even using the recommended dose has led to a reduction in bee survival in some studies [91,138,144].

Given the growing demand for more sustainable agricultural practices for pest control, ecotoxicological evaluations of biopesticides should test beyond mortality rates and assess secondary effects on the behavioral and cognitive characteristics of pollinators [142]. In three behavioral trials with *B. bassiana* on foraging bees and the species *A. mellifera* and *Tetragonisca angustula*, the following effects were verified: alteration in the nest recognition system of honey bees; alteration in the behavior and cognition of bees with inconsistent responses to sucrose; and the rejection of nest mates exposed to the fungal pathogen [134,138,142].

The pathogenicity of the fungi *B. bassiana*, *B. brongniartii*, *M. anisopliae*, *P. fumosoroseus*, and *M. brunneum* has been tested on species of springtails, aphids, ladybugs, crustaceans, lepidopteran insects, and others, with effects ranging from infection to the mortality of these non-target organisms [81,84,92,100,130]. 

In aquatic insects, *B. bassiana* was tested at lower concentrations compared to the dosages applied in the environment and impaired the development rate of insects by limiting larval growth [140]. 

It is also important to note that, in several studies, mainly with the fungi *B. bassiana* and *M. anisopliae*, no harmful effects on non-target organisms have been observed [82,97,101,103,109,110]. *M. brunneum* showed no negative impact on a predatory mite (*Gaeolaelaps aculeifer*) in the protocol developed to evaluate the lethal and sublethal effects of the fungus at recommended concentrations and ten times higher [143]. In a field study carried out in India, *B. bassiana* was found to be safe for natural enemies such as spiders, coccinellids, and lacewings [126]. In Burkina Faso, a country in Africa, a local isolate of *Metarhizium* sp. genetically developed to control the malaria vector was not harmful to bees and cockroaches [132].

### 5.3. Environment

The environment supports life, such as air, food, water for human use and consumption, climate, soil, and fuels, among others. Every human action has, to a greater or lesser extent, positive or negative impacts on the environment. Environmental health is related to the interaction between human health and the environment [185]. The concept of environmental health incorporates complex issues such as pollution, social conditions, and the assumptions of sustainable development [186]. In this context, nature conservation, good agricultural practices, and safe workplaces are prerequisites for good environmental health. Building healthier environments also includes investigating and monitoring changes in health risks and implementing solutions [185].

The long-term persistence of fungal strains released to regulate the insect population and the effect on non-target hosts and the local fungal population are concerns with regard to environmental safety (Mei et al., 2020) [85]. A field study in China selected for this review reported the characteristics of the genomic analysis and evolution of the *B. bassiana* population over 20 years following the release of exotic strains to control pine caterpillar pests. It was identified that the strains released can persist in the environment for a long time but with low recovery rates. The infection of non-target insects by the released strains occurred endemically in association with host seasonality. The *Beauveria* population evolved under balancing selection and towards expansion through adaptation, non-random interbreeding, and isolated migration [85]. 

The toxicity of the secondary metabolites produced by *M. anisopliae* was evaluated in toxicity tests with prokaryotic (bacteria) and eukaryotic organisms (protozoa, arthropods, and human cells). The study revealed that Metarhizium metabolites do not pose a risk to humans and the environment, considering that *Metarhizium* metabolites were more enriched than would ever be found in nature [148,187]. The rust fungus *Puccinia komarovii* var. glanduliferae, a candidate for classical biological control of the plant *Impatiens glandulifera*, was evaluated for its safety on other plant species in Europe. Of the 74 plant species tested, only one ornamental species was completely susceptible [149].

In work environments that use microbial agents for pest control, high concentrations of airborne microorganisms can become a problem for workers’ health. One study investigated the exposure of tomato greenhouse workers to a biocontrol product containing the fungal strain *T. harzianum* applied via drip irrigation. The results suggest that microorganisms applied by drip irrigation do not generally remain in the air in high concentrations after application. Worker exposure increased during the growing season and reached levels that could cause health symptoms during the harvest period, when there is a high demand for labor [152].

Another study assessed the viability of airborne conidia of *B. bassiana* and *M. anisopliae* indoors. The results indicated variation in conidial persistence after spray application, but it is unlikely that airborne spores from treated surfaces pose an additional risk to human health. However, considering different substrates, formulations, and abiotic factors in field environments, it is necessary to monitor changes in infectivity [151].

Fungi can use humans and other animals, protozoa, and plants as hosts [188]. The case reports of human infections in this review are of infections of fungal species used in biological pest control but are not related to the use of fungal biopesticides. Most of the reports listed here are related to the host’s immune conditions. 

In humans with immunodepression or immunosuppression, fungi are at an advantage when it comes to developing an infection [188]. Primary immunodepression is characterized by a state of an inefficient response to fight pathogens, such as genetic factors that make the individual more susceptible, from childhood to infections, and the development of autoimmune diseases and neoplasms [188].

The case reports of fungal infections found in other vertebrate animals (turtles, crocodiles, alligators, cats, and chameleons) are also not related to the use of fungal biopesticides. The environmental conditions of these animals, such as humidity and temperature, may be related to the development of fungal infections. Most of the bioassay articles are studies that analyzed the risks of possible effects on non-target organisms of the main fungal species already established in biological control (*B. bassiana* and *M. anisopliae*). The prevalence of studies on bees is due to the importance of these insects as pollinators. 

It was also possible to observe that, in many studies, the effects on non-target organisms are related to the dosage/concentration of the fungal biopesticides used in the research. Therefore, it is important to follow the recommendations for the use of any commercial biopesticide using the correct dilutions and dosages to avoid adverse effects, especially for agricultural workers. Concentration is related to the quantity of biological agents per unit volume. Thus, the higher the concentration, the greater the risk [189].

Biological pest control with microorganisms is considered a sustainable practice since it does not involve the use of chemical pesticides and therefore meets the Sustainable Development Goals (SDGs) and the integrated and unifying One Health approach. In this context, One Health, by integrating humans, animals, and the environment, can help address the full spectrum of disease control, prevention, and detection and contribute to global health security [185].

Although pest management based on fungi such as *Beauveria* and *Metarhizium* has a long history of efficacy and safety, it should not be assumed that all fungal isolates are safe [190]. In the context of One Health, health prevention and protection actions can be achieved by monitoring risk factors and activities that arise from the interaction between animals, humans, and the environment. In this sense, environmental health surveillance activities aimed at monitoring the risks of using biological pest control agents could be a reality in the future since health surveillance policies currently prioritize chemical contaminants, such as pesticides. With a worldwide trend towards the use of microorganisms for the biological control of pests, it is important to assess the risk of these biological agents on the various ecosystems in order to estimate the potential for infectious action on humans, animals, plants, or the environment.

## 6. Conclusions

This review highlights that fungus-based biopesticides represent a promising alternative to chemical pesticides, aligning with the principles of One Health. The analysis of the selected studies indicates that cases of infections in humans and other animals are not directly associated with the use of these biopesticides. However, most studies focus on bees, which is justified given their essential role in pollination. Effects on other organisms appear to depend primarily on the dosage and concentration of the biopesticides used in the research.

Nevertheless, there are still concerns regarding the long-term impacts of these biopesticides on different ecosystems and species. To ensure their safe and sustainable use, it is essential to optimize application conditions and expand research on their efficacy and potential risks across diverse environments. Future studies should also assess possible effects on exposed humans and investigate whether these fungi compete with native species, thereby preventing ecological imbalances. It is important to recognize that many of the reviewed studies are laboratory bioassays, which may overestimate fungal effects due to optimized dosing and incubation conditions. Therefore, future research should prioritize field-based ecotoxicological assessments, taking into account the complexity of natural and agricultural environments.

In summary, the adoption of fungal biopesticides can reduce environmental impacts and contribute to the safety of human, animal, and environmental health. With ongoing research and appropriate regulation, it will be possible to maximize the benefits of these products while minimizing risks and promoting their responsible use in agriculture.

## Figures and Tables

**Table 1:** Search terms for the selection of articles in the databases (Scopus, Scielo, PubMed, Web of Science, and ScienceDirect) on the subject of fungus-based bioproducts and their effects on health (human, animal, and environmental).

Search Terms
1. “biological control” AND “safety”
2. “biological control” AND “safety” AND “entomopathogenic fungi”
3. “biological control” AND “safety” AND “nematophagous fungi”
4. “entomopathogenic fungi” AND “infection”
5. “entomopathogenic fungi” AND “biocontrol”
6. “entomopathogenic fungi” AND “biosafety”
7. “entomopathogenic fungi” AND “infection” AND “safety”
8. “entomopathogenic fungi” AND “microbiology” OR “fungi” OR “safety”
9. “entomopathogenic fungi” AND “safety”
10. “entomopathogenic fungi” OR “biocontrol” AND “safety”
11. “nematophagous fungi” AND “biocontrol”
12. “nematophagous fungi”AND “safety”
13. “nematophagus fungi” AND “biological control” AND “safety”
14. “nematophagus fungi” AND “biological control”
15. “nematophagus fungi” AND “infection” AND “safety”
16. “safety” AND “biocontrol”
17. “safety” AND “biocontrol” AND “entomopathogenic fungi”
18. “safety” AND “biocontrol” AND “nematophagous fungi”

**Table 2:** Studies with fungal entomopathogens and infection/effects in humans.

Fungus	Targets	Infection/ Effects	Assessed Endpoint	References
*Beauveria bassiana*	man	Fungal keratitis	Pathogenicity, infectivity	[26]
*Metarhizium anisopliae*	man	Fungal keratitis	Pathogenicity, infectivity	[27]
*Trichoderma longibrachiatum*	woman	Invasive sinusitis	Pathogenicity	[28]
*Metarhizium anisopliae*	child	Skin lesions	Pathogenicity	[29]
*Metarhizium anisopliae*	man and woman	Invasive sinusitis	Pathogenicity	[30]
*Beauveria bassiana*	woman	Fungal keratitis	Pathogenicity, infectivity	[31]
*Metarhizium anisopliae*	woman	Fungal keratitis	Pathogenicity, infectivity	[32]
*Beauveria bassiana ou Beauveria brongniartii*	woman	Systemic fungal infection	Pathogenicity, infectivity	[33]
*Beauveria bassiana*	woman	Skin lesions, respiratory symptoms, and pleural effusion	Pathogenicity	[34]
*Akanthomyces lecani*	216 women and 113 men	High prevalence of sensitization	Allergenicity	[35]
*Metarhizium anisopliae*	allergic patients	Positivity to the allergenicity test with atopic individuals from the sugar cane area (29%), atopic individuals from urban areas (95%)	Allergenicity	[22]
*Beauveria bassiana*	patients with hypersensitivity to fungi	Allergenic potential	Allergenicity	[23]
*Beauveria bassiana*	man	Empyema	Pathogenicity	[36]
*Beauveria bassiana*	serum from patients with fungal allergies	Four alleged allergens identified	Allergenicity	[24]
*Beauveria bassiana*	woman	Fungal keratitis	Pathogenicity, infectivity	[37]
*Metarhizium anisopliae*	woman	Sclerokeratitis	Pathogenicity	[38]
*Beauveria bassiana*	woman	Fungal keratitis	Pathogenicity, infectivity	[39]
*Metarhizium anisopliae*	child	Skin lesions	Pathogenicity	[40]
*Beauveria bassiana*	woman	Necrotizing scleritis and endophthalmitis	Pathogenicity	[41]
*Beauveria bassiana*	woman	Fungal keratitis	Pathogenicity, infectivity	[42]
*Beauveria bassiana*	human lymphocytes	Beauvericin (BEA) is genotoxic to human lymphocytes in vitro	Toxicity	[25]
*Metarhizium anisopliae*	child	Fungal keratitis	Pathogenicity, infectivity	[43]
*Beauveria bassiana*	woman	Bullous keratopathy	Pathogenicity	[44]
*Trichoderma longibrachiatum*	woman	Rhinosinusitis	Pathogenicity	[45]
*Beauveria bassiana*	woman	Fungal keratitis	Pathogenicity, infectivity	[46]
*Beauveria bassiana*	man	Fungal keratitis	Pathogenicity, infectivity	[47]
*Metarhizium anisopliae*	homem	Fungal keratitis	Pathogenicity, infectivity	[48]
*Beauveria bassiana*	woman	Fungal keratitis	Pathogenicity, infectivity	[49]
*Metarhizium anisopliae*	man	Sclerokeratitis	Pathogenicity	[50]
*Beauveria bassiana*	woman	Fungal keratitis	Pathogenicity, infectivity	[51]
*Metarhizium anisopliae*	woman	Fungal keratitis	Pathogenicity, infectivity	[52]
*Metarhizium anisopliae*	woman	Sclerokeratitis and endophthalmitis	Pathogenicity	[53]
*Metarhizium anisopliae*	woman	Fungal keratitis	Pathogenicity, infectivity	[54]
*Beauveria bassiana*	man	Fungal keratitis	Pathogenicity, infectivity	[55]

**Table 3:** Studies with fungal entomopathogens and their respective effects on vertebrate animals.

Fungi	Vertebrate Animals	Effects	References
*Beauveria bassiana Cordyceps fumosorosea* (*Paecilomyces fumosoroseus)*	Turtles (*Testudo elephantopus*, *Testudo gigantea elephantina,* and *Terrapene carolina*)	Pulmonary infection	[56]
*Beauveria bassiana*	American alligator (*Alligator mississippiensis*)	Pulmonary lesions	[57]
*Cordyceps fumosorosea* (*Paecilomyces fumosoroseus)*	murine	No effects	[61]
*Metarhizium anisopliae Cordyceps fumosorosea (Paecilomyces fumosoroseus)*	Japanese quails (*Coturnix coturnix japonica*)	Loss of body weight	[62]
*Beauveria bassiana Metarhizium anisopliae Metarhizium rileyi* (*Nomuraea rileyi*) *Cordyceps farinosa (Paecilomyces farinosus) Cordyceps fumosorosea* (*Paecilomyces fumosoroseus) * *Neoconidiobolus thromboides (Entomophthora virulenta)*	Birds, rats, and frogs	Temporary changes in the stomach and intestines	[63]
*Hirsutella thompsonii*	Rats, rabbits, and guinea pigs	No effects	[64]
*Metarhizium anisopliae*	Rats	Tissue damage	[65]
*Lagenidium giganteum*	Mice, rats, and rabbits	The fungus can persist in mammalian tissues	[66]
*Beauveria bassiana*	Fish embryos (*Menidia beryllina*)	Embryo rupture and death were observed	[67]
*Beauveria bassiana*	Turtle (*Trachemys scripta*)	Pulmonary infection	[58]
*Metarhizium anisopliae*	Cat	Invasive rhinitis with spread of infection to the nasal bones and subcutaneous tissues	[59]
*Metarhizium anisopliae var. acridum Beauveria bassiana*	Ring-necked pheasants (*Phasianus colchicus*)	No effects	[68]
*Metarhizium anisopliae*	Fishes (*Melanotaenia duboulayi, Ulmerophlebia* spp., and *Ceriodaphnia dubia*)	100% mortality of *Ceriodaphnia dubia* in 48 h	[69]
*Metarhizium anisopliae*	Lizard (*Acanthodactylus dumerili)*	Death of 2 lizards and liver changes	[70]
*Cordyceps fumosorosea* (*Paecilomyces fumosoroseus)*	Mice	No permanent damage to liver tissue	[71]
*Metarhizium anisopliae*	BALB/c Mice	Increased allergic response, weight and number of popliteal lymph node cells	[72]
*Metarhizium anisopliae*	BALB/c Mice	Potential for allergy induction	[73]
*Metarhizium anisopliae*	American alligator (*Alligator mississippiensis*)	Fungal pneumonia, anorexia, and abnormal buoyancy	[60]
*Trichoderma stromaticum*	BALB/c Mice	Immunosuppressive effect	[74]
*Cordyceps tenuipes* (*Paecilomyces tenuipes*)	Mice and rats	No toxicological effects	[75]
*Cordyceps fumosorosea* (*Isaria fumosorosea*)	Rabbits	No toxic effects	[76]
*Metarhizium viride*	Veiled chameleons (*Chamaeleo calyptratus*), panther chameleons (*Furcifer pardalis*), and lizards (*Pogona vitticeps*)	Fungal glossitis, stomatitis, pharyngitis, or visceral mycosis	[77]
*Isaria cicadae* (*Cordyceps cicadae*)	Mice	No effects on the CNS or cardiovascular and respiratory systems	[78]
*Beauveria bassiana*	Albino rats	Effect on liver function and size	[79]
*Aspergillus parasiticus*	Fish (*Hypophthalmichthys molitrix*)	Cracked eyes (64%), bleeding in the fins (33%), and infection in the scales (30%)	[80]

**Table 4:** Studies with fungal entomopathogens and their respective effects on invertebrate animals.

Fungi	Invertebrate Animals	Effects	References
*Hirsutella thompsonii*	*Apis mellifera*	No effects	[86]
*Entomophaga maimaiga * *Beauveria bassiana*	*Apis mellifera*	*E. naimaga* did not affect the longevity of the bees. *B. bassiana* reduced longevity and caused mycosis in bees at higher concentrations	[87]
*Beauveria bassiana Metarhizium anisopliae* *Cordyceps fumosorosea* (*Paecilomyces fumosoroseus) * *Paecilomyces farinosus*	Earthworm cocoons (*Aporrectodes caliginosa*)	The fungi did not reduce the hatching rate of the cocoons	[88]
*Nosema meligethi I.*	*Apis mellifera*	*Apis mellifera* showed no sign of infection after 30 days	[89]
*Metarhizium anisopliae Beauveria bassiana*	*Apis mellifera*	Almost all the bees died at the higher concentration	[90]
*Metarhizium flavoviride*	*Apis mellifera*	A field dose formulated in oil killed 11% of the bees and a similar dose formulated in water killed 8%	[91]
*Beauveria bassiana*	Shrimp (*Palaemonetes pugio*)	Lethal infections when conidiospores were injected	[92]
*Metarhizium anisopliae*	Beetles, grasshoppers, and butterflies	There were no differences in the number of late-stage larvae of the non-target species	[93]
*Metarhizium rileyi* (*Nomuraea rileyi*) *Beauveria bassiana Metarhizium anisopliae*	*Apis mellifera*	*B. bassiana* and *M. anisopliae* caused greater mortality in the bees’ liquid diet	[94]
*Metarhizium anisopliae*	Shrimp (*Palaemonetes pugio*)	Dead embryos and larvae with fungal growth and delayed embryo hatching	[95]
*Akanthomyces lecani*	20 non-target invertebrates	There was no evidence of infection in non-target species	[96]
*Metarhizium anisopliae Beauveria bassiana*	Beetles (*Pimelia senegalensis; Trachyderma hispida*)	No infection was observed	[97]
*Beauveria bassiana*	Oribatida (scarab mites)	Reduction in the abundance of Oribatida	[98]
*Metarhizium flavoviride Beauveria bassiana*	Beetle (Malagasy Coleoptera)	*B. bassiana* had no impact on the number of species. *M. flavoviride* had a significant impact on non-target beetles	[99]
*Cordyceps fumosorosea* (*Paecilomyces fumosoroseus)*	Aphid (*Diuraphis noxia*) ladybug (*Hippodamia convergens*)	Infection of predators (aphids and ladybugs)	[81]
*Beauveria bassiana Beauveria brongniartii * *Metarhizium anisopliae*	Three species of collembola (*Folsomia fimetaria; Hypogastrura assimilis; Proisotoma minuta*)	Low virulence for non-target springtails	[100]
*Metarhizium anisopliae*	Epigean arthropods	No evidence of infection of native non-target arthropods in field conditions	[101]
*Beauveria bassiana*	Insect cadaver samples (non-target hosts)	Released strains of *B. bassiana* can be isolated from non-target insects	[102]
*Beauveria brongniartii*	*Poecilus versicolor*	No negative effects were observed	[103]
*Beauveria bassiana*	*Apis mellifera*	No adverse effects on bees	[104]
*Trichoderma viride Trichoderma* spp. *Clonostachys rosea f. catenulata* (*Gliocladium catenulatum*) *Trichoderma virens* (*Gliocladium virens*) *Metarhizium anisopliae Beauveria brongniartii Stagonospora* spp.	Crustaceans (*Artemia salina* and *Daphia magna*)	Both invertebrates were very sensitive to all the metabolites examined	[105]
*Beauveria bassiana*	*Bombus impatiens*	The average concentration was considered ideal with the least impact on bees	[106]
*Beauveria bassiana Metarhizium anisopliae*	Natural enemies (*Coccinella septempunctata* L.; *Chrysoperla carnea; Dicyphus* *tamaninii*) and beneficial insect (*Heteromurus nitidus*)	*B. bassiana* and *M. anisopliae* are relatively safe for non-target insects	[107]
*Trichoderma harzianum Trichoderma polysporum*	*Bombus terrestris*	The biofungicides did not cause any bee mortality	[108]
*Beauveria bassiana*	*Apis mellifera*	Did not affect the health of the bee colony	[109]
*Beauveria bassiana Metarhizium anisopliae Akanthomyces lecani Hirsutella kirchneri Hirsutella nodulosa Hirsutella* sp.	*Apis mellifera*	Adult bees and pupae were not susceptible to the fungal isolates tested	[110]
*Trichoderma harzianum * *Beauveria bassiana*	*Bombus terrestris*	*B. bassiana* caused 92% mortality after 11 weeks of exposure; *T. harzianum* caused no negative effect	[111]
*Metarhizium anisopliae*	*Apis mellifera*	Higher bee mortality with conidial spraying	[112]
*Arcopilus cupreus* (*Chaetomium cupreum*)	*Artemia salina*	Toxicity caused by the secondary metabolite oosporein	[113]
*Beauveria bassiana Metarhizium anisopliae Akanthomyces lecani*	*Apis mellifera*	Higher mortality of bees treated (feeding) with *V. lecanni* and *B. bassiana*	[114]
*Beauveria bassiana*	Mantis (*Tenodera sinensis; Statilia maculate*)	Epizootic in mantis populations in a vast region of China (panzootia) in 2009	[115]
*Beauveria bassiana*	*Megachile rotundata Apis mellifera*	Susceptibility (mortality) of bees dependent on fungal concentration	[116]
*Beauveria bassiana*	*Bombus impatiens*	Oral or topical application of *B. bassiana* had no effect on bees	[117]
*Beauveria bassiana*	*Apis mellifera*	Toxic to worker larvae	[118]
*Metarhizium anisopliae Metarhizium robertsii*	Bed bugs (*Schiodtella formosana*)	Causative agents of epizootic green muscardine disease in populations of *S. formosana*	[119]
*Metarhizium anisopliae*	*Bombus terrestris*	Dry exposure to *M. anisopliae* spores caused mortality	[120]
*Beauveria bassiana*	*Melipona scutellaris*	Highly virulent (bee mortality at lower dose)	[121]
*Beauveria bassiana*	Silkworm (*Bombyx mori*)*,* Pine caterpillar (*Dendrolimus punctuatus*)	The fungal infection did not originate from the fungal insecticide	[122]
*Clonostachys rosea f. catenulata* (*Gliocladium catenulatum*) *Beauveria bassiana*	*Bombus terrestris*	Cuticular water loss and reduced survival	[123]
*Beauveria bassiana*	*Amblyseius swirskii* (predatory mite)	*A. swirskii* was susceptible to *B. bassiana* when the conidia were applied directly	[124]
*Metarhizium brunneum Beauveria bassiana*	*Trybliographa rapae*	Low risk to *T. rapae* populations (parasitoid)	[125]
*Beauveria bassiana*	Natural enemies: spiders, coccinellids, and lacewings	No effects on natural enemies	[126]
*Metarhizium anisopliae*	1944 non-target arthropod species	Mortality of small numbers of non-target insects living in the soil	[127]
*Beauveria bassiana * *Metarhizium anisopliae* *Isaria fumosorosea*	Stingless bees (*Tetragonisca angustias Latreille, Scaptotrigona mexicana Guérin-Meneville,* and *Melipona beecheii Bennett*)	Moderate to high impact on bee mortality	[128]
*Beauveria bassiana*	Phytoseiid mites (*Neoseiulus cucumeris*; *N. californicus*; *Phytoseiulus persimilis*; *N. womersleyi*; *Amblyseius swirskii*)	No effect on predatory mites	[82]
*Beauveria bassiana*	*Apis mellifera*, *Chrysoperla rufilabris;Orius insidiosus; Hippodamia convergens; Harmonia axyridis;Coleomegilla maculata* and *Aranea*	Low lethality at low doses	[129]
*Metarhizium brunneum*	*Galleria mellonella*	Mortality of larvae and cadavers with low fungal growth	[130]
*Clonostachys rosea f. catenulata* (*Gliocladium catenulatum*) *Beauveria bassiana Metarhizium brunneum*	*Apis mellifera* *Bombus terrestris*	Effects on the longevity of the two bee species	[131]
*Metarhizium* spp.	American cockroaches (*Periplaneta americana*), bees (*Apis mellifera adansonii)*	There was no significant increase in mortality and no mycosis was observed on the cadavers	[132]
*Beauveria bassiana Metarhizium anisopliae*	*Apis mellifera*	Reduced worker survival	[133]
*Beauveria bassiana*	Foraging bees	Changes to the nest recognition system	[134]
*Beauveria bassiana Akanthomyces lecani Metarhizium anisopliae*	*Apis mellifera Bombus terrestris*	Slight effect on bee movement and death of some individuals	[135]
*Aspergillus flavus*	Crustacean (*Alpheus bouvieri*); mosquito (*Toxorhynchites splenden*)	80% mortality of both species	[84]
*Beauveria bassiana Metarhizium rileyi* (*Nomuraea rileyi*)	*Apis cerana*	*B. bassiana* was slightly to moderately toxic to bees. *Metarhizium rileyi* (*Nomuraea rileyi*) was harmless to bees	[136]
*Beauveria bassiana Metarhizium anisopliae*	*Eudrilus eugeniae*	Little impact on earthworm mortality	[83]
*Beauveria bassiana*	*Apis cerana*	No effect on bees. Temperature inside the hive at 35 °C abolished conidia germination	[137]
*Beauveria bassiana*	*Apis mellifera*	Effect on behavior and cognition (inconsistent response to sucrose)	[138]
*Beauveria bassiana Metarhizium anisopliae*	Africanized *Apis mellifera* queens	Reduced worker survival	[138]
*Beauveria bassiana*	Africanized *Apis mellifera*	Increase in weight and time of bee emergence	[139]
*Beauveria bassiana*	Aquatic insects (*Chironomu riparius*)	Lethal and sublethal effects and on larval growth	[140]
*Beauveria bassiana*	Non-target insects	Released strains can infect non-target insects	[85]
*Metarhizium anisopliae Beauveria bassiana*	*Apis meelifera Melipoluna ferruginea*	Reduced survival of *Apis mellifera*	[141]
*Beauveria bassiana*	*Tetragonisca angustula*	Change in behavior	[142]
*Metarhizium brunneum*	Predatory mite *Gaeolaelaps* (*Hypoaspis*) *aculeifer*	No negative impact	[143]
*Beauveria bassiana Metarhizium anisopliae Cordyceps fumosorosea (Paecilomyces fumosoroseus)*	Bees (*Tetragonisca angustula*; *Scaptotrigona depilis*; *Apis melífera Bombus terrestris*)	Reduced bee survival	[144]

**Table 5:** Studies with fungal entomopathogens and their respective effects on vertebrate animals, invertebrates, plants, and other organisms.

Fungus	Organisms	Effects	References
*Lagenidium giganteum*	*Apis mellifera*; microcustaceans (decapods, copepods, ostracods); quail (*Colinus virginianus* L.); ducks, fish, insects, plants, and algae	Two species of insects were infected by exposure to the fungus	[145]
*Lagenidium giganteum*	Cladocerans (*Ceriodaphnia dubia, Daphnia pulex, D. magna*) and fish (*Pimephales promelas)*	*L. giganteum* can affect some non-target aquatic species	[146]
*Metarhizium anisopliae*	Embryos of grass shrimp (*Palaemonetes pugio*), kingfishers (*Menidia beryllina*), and frogs (*Xenopus laevis*)	Eye abnormalities in shrimp and frog embryos. Frog embryos with moderate to severe cranial, facial, and intestinal malformations. Significant mortality in grass shrimp and kingfish embryos	[147]
*Metarhizium anisopliae*	Bacterium (*Pseudomonas syringae*), protozoan (*Tetrahymena pyriformis*), arthropod (*Daphnia magna*), human cell line and an insect cell line (*Spodoptera frugiperda)*	Toxicity to insect cells. Secondary metabolites without risk to humans or the environment	[148]
*Puccinia komarovii var. glanduliferae*	74 plant species tested	An ornamental species was susceptible (*Impatiens balsamina*)	[149]
*Aspergillus flavus*	*Spodoptera litura* and rats	No toxicity to mammals	[150]

**Table 6:** Studies evaluating airborne exposure to fungal entomopathogens and toxicity of secondary metabolites.

Fungus	Test	Toxicity	Reference
*Metarhizium anisopliae Beauveria bassiana*	Viability of airborne conidia	No risk to humans	[151]
*Trichoderma harzianum*	Bioaerosol analysis	*T. harzianum* was detected in the air only on the day of the treatment	[152]
*Beauveria brongniartii*	Physico-chemical analysis	It is unlikely that the metabolite oosporein can be absorbed into organisms and pass through the biological membrane	[153]
*Beauveria bassiana Metarhizium anisopliae Beauveria brongniartii Albifimbria verrucaria* (*Gliocladium fimbriatum*)	Ames Test and VITOTOX	No genotoxicity of secondary metabolites was observed: oosporein, gliotoxin, and destruxins B, D, and E	[154]

## Data Availability

The datasets used and analyzed during this study are available from the corresponding author upon reasonable request.

## Supplementary Materials

The following supporting information can be downloaded at: https://www.mdpi.com/article/10.3390/pathogens14050463/s1, Table S1: PRISMA checklist.
